# Exploring actual and perceived levels of physical activity intensity during virtual reality active games

**DOI:** 10.3389/fspor.2024.1349521

**Published:** 2024-02-09

**Authors:** Keith E. Naugle, Xzaliya A. Cervantes, Carolyn L. Boone, Brandon Wind, Kelly M. Naugle

**Affiliations:** ^1^ Department of Kinesiology, School of Health and Human Sciences, Indiana University Purdue University Indianapolis (IUPUI), Indianapolis, IN, United States; ^2^ College of Osteopathic Medicine, University of Pikeville, Pikeville, KY, United States

**Keywords:** virtual reality, active gaming, perceived exertion, physical activity, exercise intensity

## Abstract

**Background:**

Research suggests that engaging in active virtual reality (VR) video games can elicit light to moderate levels of physical activity (PA), making it a novel and fun mode of exercise. Further research is needed to understand the influence of VR on perceptions of exertion and enjoyment during PA.

**Objective:**

The objectives of this study are (1) to compare actual and perceived exertion within and between active VR games with varying levels of difficulty and (2) to determine how playing active VR games influences PA enjoyment during gameplay.

**Methods:**

A total of 18 participants completed four separate study sessions, during which they engaged in either a 15-min bout of traditional exercise (stationary cycling) or played one VR game. Heart rate (HR) and ratings of perceived exertion (RPE) using the Borg CR10 scale were assessed during VR gameplay and cycling. Enjoyment was measured after gameplay. VR games included playing Holopoint at level 2 and level 3 and Hot Squat. Repeated measures ANOVAs were used to examine (1) changes in HR and RPE across time within games and (2) differences in actual and perceived levels of intensity and enjoyment between games. Bivariate correlations examined the relationship between the degree of change in actual intensity and the degree of change in perceived intensity during each VR game and cycling.

**Results:**

The analyses revealed that RPE and HR significantly increased from baseline during each condition and generally increased across the 15-min of gameplay. Hot Squat and cycling elicited a significantly higher percentage of heart rate reserve (%HRR) than Holopoint at levels 2 and 3. Holopoint level 3 elicited a higher %HRR than Holopoint level 2. The participants reported greater average and max RPE during Hot Squat and cycling compared with Holopoint at levels 2 and 3. The correlations revealed a significant positive correlation between the degree of change in HR and RPE for cycling, but no significant correlations were observed for any of the VR conditions. The physical activity during Holopoint at both levels was rated as more enjoyable than Hot Squat and cycling.

**Conclusion:**

Our data support the notion that VR has the potential to alter individuals’ perceptions of exertion during PA and, in particular, may reduce their awareness of increases in actual exertion.

## Introduction

Video games have emerged as a primary source of entertainment worldwide. Over 200 million people play video games in the United States, with approximately 40% of that group between the ages of 18 and 35 years old (1). In the past decade, virtual reality (VR) gaming has significantly grown in popularity, with approximately 30% of gamers now owning a VR device (1). VR consoles typically include motion-tracking hand controllers and a headset that displays a fully immersive 3D environment. Player movements are tracked via the controllers and headset, which allow interactions between the player and virtual objects in the 3D environment. Many recent commercial VR video games have been released that require significant movement during gameplay (i.e., Holopoint, Beat Saber). Recent research has evaluated whether active VR games elicit physical activity that can count towards the recommended 150 min of moderate to vigorous aerobic exercise per week (2–5). The results of these studies are mixed and potentially dependent on the type of game and its difficulty level. Finding new and enjoyable modes of exercise is important due to the fact that approximately 40%–50% of young to middle-aged adults do not meet the recommendations for aerobic exercise (6).

The parallel processing model of attention theorizes that attentional strategies can affect the judgment of sensory cues, with dissociative strategies capable of decreasing perceptions of exertion during physical activity (7). Indeed, with a dissociative strategy during exercise, an individual focuses on external cues (e.g., auditory and visual stimuli in the environment) not related to the exercise, thereby providing a distraction from internal sensations. Thus, a potential benefit of exercising via active VR games is that the VR environment could facilitate the use of dissociative attentional strategies and could provide a positive distraction from unpleasant bodily symptoms that arise during higher-intensity physical activity (8). While the research is mixed, several studies have shown that active VR games have the potential to elicit moderate-intensity physical activity while keeping perceived effort lower during gameplay (3, 5). For example, Gomez et al. (3) demonstrated that active VR games, including Holopoint, had higher categorizations of physical activity intensity via objective measures (metabolic equivalents: METS) compared with perceived exertion intensity measures (RPE). Holopoint was perceived as light intensity even though it fell within the moderate category as defined by METs. However, Evans et al. (2) reported similar intensity categorizations based on RPE and percentage of heart rate reserve (%HRR) for VR games Beat Saber, Holopoint, and Hot Squat, with only Hot Squat reaching moderate intensity. Most recently, Stewart et al. (5) revealed that participants' perceptions of exertion were less than their actual exertion when playing the active VR games Fruit Ninja VR, Beat Saber, and Holopoint. A limitation in the Evans and Stewart studies is that actual exertion was measured during gameplay, while perceived exertion was measured after gameplay. Other limitations of prior studies included a lack of an exercise-only control condition (2, 3, 5), implementing a VR environment on a 2D screen (5), and the use of relatively short durations of gameplay (e.g., Gomez and Stewart’s studies analyzed only 4–5 min of gameplay). In addition, no studies have evaluated whether participants accurately perceive changes in exertion while playing VR games. Thus, more research is needed to fully understand the influence of VR on perceptions of exertion during physical activity. This is important because prior research has shown that perceived exertion during physical activity can impact adherence to physical activity programs (9).

The current study was designed to address many of the aforementioned limitations by (1) measuring actual and perceived exertion during gameplay over a relatively longer duration, (2) evaluating whether changes in exertion are accurately perceived during VR, and (3) including an exercise control condition. Thus, the overall purpose is to determine whether changes (within a game) and differences (between games) in actual exertion correspond to changes and differences in perceived exertion during VR games with varying levels of difficulty. A secondary purpose is to determine how playing active VR games influenced physical activity enjoyment during gameplay compared with traditional exercise matched for aerobic intensity. Prior research on active VR gaming has evaluated the level of enjoyment but rarely compared it with traditional exercise. Heart rate (HR: actual exertion) and ratings of perceived exertion (RPE) using the Borg CR10 scale were assessed during 15 min of VR gameplay and traditional exercise. VR games included playing Holopoint, at different difficulty settings (level 2 vs. level 3) in separate sessions, and Hot Squat. Holopoint is a game that uses the upper and lower body to dodge incoming targets and hit targets with a bow and arrow. Hot Squat is primarily a lower-body game that requires squatting to avoid incoming objects. The physical difficulty of Hot Squat increases progressively throughout the game. Based on the results of the study conducted by Evans et al. (2), we hypothesized that (1) Hot Squat would elicit higher levels of perceived and actual exertion compared with Holopoint and (2) that playing Holopoint at level 3 would elicit higher actual exertion compared with Holopoint at level 2. Regarding the changes in exertion within a game, we hypothesized that actual and perceived exertion would increase from baseline and across time for all VR games and exercises. However, we also hypothesized that the degree of change in actual intensity would be more strongly associated with the degree of change in perceived intensity during traditional exercise compared with active VR games. This hypothesis is based on the notion that we expect participants to engage in more dissociative strategies during VR compared with traditional exercise (8), thereby leading to less accurate perceptions of exertion.

## Materials and methods

### Participants

The study included a total of 21 participants (11 males, 10 females) aged between 18 and 34. All participants completed an IRB-approved informed consent form prior to study participation. The participants were recruited from the local university with posted study flyers. The exclusion criteria included (1) motion sickness or claustrophobia, (2) an acute or chronic pain condition, and (3) an answer of “yes” on any of the general health questions on the Physical Activity Readiness Questionnaire (PAR-Q+2019 version) (10). The study session exclusion criteria included eating the hour before each session, consuming alcohol within 24 h of the sessions, participating in vigorous exercise on the day of the sessions prior to the session, and ingesting caffeine or analgesic medications on the day of the sessions prior to the session.

### Procedures

This study utilized a repeated measures design in which the participants completed all procedures and conditions. The participants completed five sessions on separate days. The current study is part of a larger study on active gaming and will only include a description of the methods and data relevant to the current study. This study was approved by the Indiana University Institutional Review Board.

#### Enrollment, screening, and familiarization (beginning of Session 1)

At the beginning of the first study session, the participants signed a written informed consent and completed the PAR-Q+ and a demographics questionnaire to verify eligibility. Then, the participants were familiarized with the Meta Quest 2 VR system (Menlo Park, CA), which includes a headset and two handheld controllers. The VR system tracks the movements of the head and controllers and then translates these movements into the 3D environment displayed on the headset. At the start of Session 1, the participants also completed the International Physical Activity Questionnaire—Short Form (IPAQ-SF) (11) and sat quietly for 10 min to measure their resting heart rate.

#### Experimental sessions 1–5

Excluding the informed consent, screening, and familiarization procedures in Session 1, the procedures for Sessions 1 through 5 were identical except for the type of activity completed during each session. The participants played one VR game during sessions 1–4, which included Holopoint, Hot Squat, and Relax Walk. Holopoint was played in two separate sessions with one session played at level 2 (L2) and the other at level 3 (L3). Relax Walk is a stationary game, and therefore the session including this game was not included as a part of this study (Relax Walk was part of the larger study). The order of games during sessions 1–4 was randomized. All VR games were played in a 6.5 × 8.5 feet space. See Table 1 for the description of the games. Traditional exercise in the form of stationary cycling was completed during Session 5.

**Table 1:** Description of VR games and exercise.

Condition	Description
Holopoint (VR)	Holopoint is a fast-paced archery game in which participants use the controllers as a bow and arrow to hit incoming targets. When the targets are hit, players must dodge the projectiles that fire back at the player. The speed and volume of targets increase with higher levels.
Hot Squat (VR)	Hot Squat is a squatting game to music that requires participants to continually perform squats, and sometimes hold a squat, to avoid incoming objects.
Stationary cycling (non-VR)	Participants rode a stationary bicycle at a predetermined intensity. Participants adjusted speed of cycling to adjust intensity.

**Figure 1:**
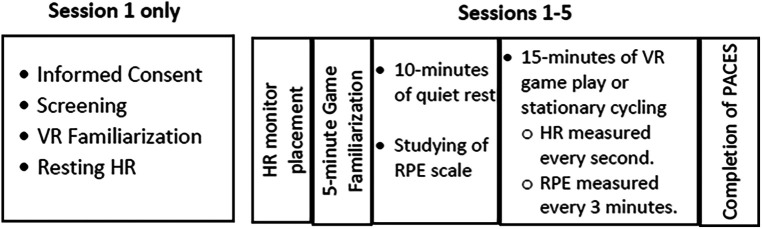
Order of experimental events. VR, virtual reality; HR, heart rate; RPE, ratings of perceived exertion.

The order of experimental events is depicted in Figure 1. Prior to gameplay, the participants were fitted with a Polar HR monitor consisting of an HR sensor placed around the chest and a wristwatch placed on the non-dominant wrist. Then, the participants played the assigned game or rode the stationary bike at a very light intensity for 5 min for familiarization and then sat quietly for 10 min to allow HR to return to rest. Next, the participants played the assigned game or rode the bike for 15 min. During Session 5, the intensity of the stationary cycling was matched to the intensity (based on HR) of the highest intensity played during VR gameplay. For example, if a participant played Hot Squat at the highest intensity based on HR, then the average HR during Hot Squat for minutes 1–5, 5–10, and 11–15 was determined for that participant. If the average HR for Hot Squat for minutes 1–5 was 115 beats, then a target HR range of 110–120 (average ±5) beats was created for minutes 1–5 on the bike. The experimenter instructed the participant to bike faster or slower to keep their HR within the target range. During each 15-min bout of gameplay or exercise, HR was continuously monitored, and RPE using the Borg CR10 scale was assessed every 3 min. The modified Physical Activity Enjoyment Scale (PACES) was completed after the 15-min bout of activity.

### Outcome measures

#### Measures of actual exertion

A Polar HR monitor (Polar, Kempele, Finland) was used to measure HR every second during gameplay. The HR measured at baseline (just prior to starting the activity) and during the middle 13 min (i.e., excluding the first and last minute) of the 15-min period was used for data analyses. Raw HR values were averaged for minutes 1–4, 5–9, and 10–14. The maximum HR value and average HR value for minutes 2–14 were also recorded. Max HR was determined by the maximum HR value recorded during minutes 2–14 of gameplay. The max and average HR values were used to calculate the percentage of max and average HR reserve (%HRR) values for each game and exercise. The percentage of HRR was calculated with the following formula: [(HR during activity − resting HR)/HRR] × 100, with HRR = maximum age-related HR − resting HR. The maximum age-related HR was calculated with the standard formula of 220 − age. The %HRR ranges that were used to determine physical activity intensity were the following: light: 30%–39%, moderate: 40%–59%, and vigorous: ≥60%) (12, 13). We also calculated the percentage of time that the participants were in moderate to vigorous intensity during the 15-min bout (i.e., %HRR values that were ≥40% for each second of gameplay). In sum, the following measures were extracted from HR to represent actual exertion: average %HRR, max %HRR, and percent of time in moderate to vigorous physical activity (MVPA).

#### Measure of perceived exertion

During the 15-min bout, the participants were asked to rate their exertion levels using the 0–10 Borg Category-Ratio scale (Borg CR10), where 0 indicates “nothing at all” and 10 represents “extremely strong—Maximal” (14). Specifically, the participants were instructed, “When rating exertion give a number that corresponds to how hard and strenuous you perceive the activity to be. The perception of exertion is mainly felt as strain and fatigue in your muscles and as breathlessness.” The participants were also told that it is important to report what they actually experience or feel, not what they think they should report (14). During the 10 min of quiet rest between familiarization and the 15-min activity bout, the participants were given the Borg CR10 scale to study since they would be asked to give ratings without viewing the scale. The participants were asked to give RPE ratings at baseline and 3, 6, 9, 12, and 15 min of the 15-min bout while still wearing the VR headset. Thus, the participants could not see the scale while giving a rating. To mimic the VR sessions, the participants also did not have access to the scale during stationary cycling. The average RPE and maximum RPE were calculated based on the five ratings provided during gameplay or exercise.

#### Enjoyment

Upon the completion of each active game, the participants were asked to complete the modified Physical Activity Enjoyment Scale (PACES). The PACES includes five Likert-style questions related to enjoyment of the activity. The PACES questionnaire consisted of items rated on a seven-point scale, assessing perceived feelings that ranged from (1) enjoy to hate, (2) dislike to like, (3) fun to no fun, (4) feel good physically to feel bad physically, and (5) frustrated to not frustrated. The participants were instructed to rate how they felt about the physical activity they had recently engaged in. Each question had a maximum score of seven. The percentage of the sum of the individual questions out of 35 for PACES was used in the statistical analysis, with higher scores indicating greater enjoyment. The PACES has been used in prior active gaming studies and is a validated tool (15–18).

### Statistical analysis

Statistical analyses were conducted with SPSS v29 (IBM Corporation, Armonk, NY). Descriptive characteristics were calculated for all primary variables. Raw HR values were averaged for minutes 1–4, 5–9, and 10–14. We conducted a 4 (Condition) ×  4 (time: baseline, 1–4 min, 5–9 min, 10–14 min) repeated measures ANOVA to examine changes across time in HR for each condition. Similarly, we conducted a 4 (condition) ×  6 (time: baseline, 3, 6, 9, 12, 15 min) repeated measures ANOVA to examine the changes across time in RPE for each condition. To examine the differences in the actual exercise intensity between games, the percentage of time in MVPA, average %HRR, and max %HRR were analyzed with separate one-way repeated measures ANOVAs. To examine the differences in perceived intensity and enjoyment between games, the average RPE, max RPE, and PACES scores were analyzed with separate one-way repeated measures ANOVAs. *Post-hoc* analyses were conducted using simple effects tests for analyzing significant interactions, and *t*-tests with Bonferroni corrections were conducted for assessing significant main and simple effects.

We also examined whether the degree of change (within a game) in actual intensity correlated with the degree of change in perceived intensity during each VR game and stationary cycling. The degree of change was evaluated by calculating the slope of the change in intensity from minute 3 to minute 12 in %HRR and RPE. The average %HRR was calculated for minute 3 and minute 12 of gameplay and stationary cycling. Minutes 3 and 12 were chosen because we expected the participants to be in a steady state during these time points. The slope of the line representing a change in intensity from minute 3 to minute 12 was calculated with the following formulas: slope for RPE = (minute 12 RPE − minute 3 RPE)/(12 − 3) or slope for %HRR = (minute 12%HRR − minute 3%HRR)/(12 − 3). Bivariate correlations were conducted between the %HRR slope and RPE slope for each VR game and cycling. Significance was set at *p* < .05.

## Results

Of the 21 participants enrolled in this study, a total of 18 participants completed all of the required conditions (11 males, average age = 23.8 years, SD = 4.7) and were included in the data analyses. The data of two participants were excluded due to the inaccurate collection of HR measurements during one of the games. Another participant's data was excluded due to their failure to complete the stationary cycling session. The average IPAQ-SF total score was 4,861.75 ± 3,693.9, indicating that the participants in the sample were highly physically active.

### Changes across time in HR and RPE between conditions

#### Heart rate

**Figure 2:**
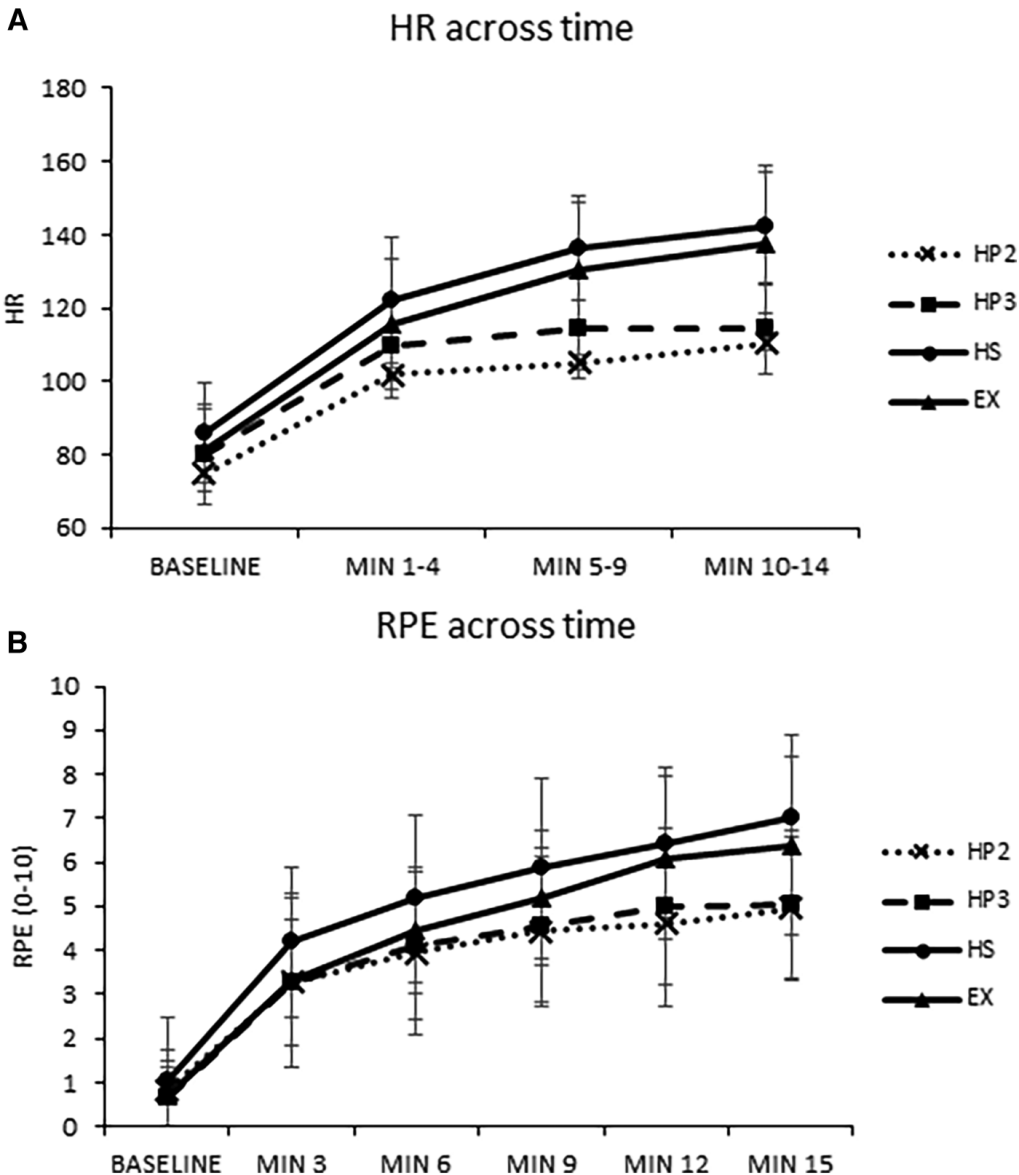
(**A**) Heart rate (HR) across time for each condition; (**B**) ratings of perceived exertion (RPE) across time for each condition. Min, minutes; HP2, Holopoint level 2; HP3, Holopoint level 3; HS, Hot Squat; EX, exercise (stationary cycling).

The two-way ANOVA revealed a main effect of condition (*p* < .001) and time (*p* < .001), which were superseded by a significant interaction, *p* < .001. The simple effects tests of time within each condition were significant (*p* < .001). The significant follow-up tests revealed the following differences across time: (1) All games increased HR from baseline to each time point, (2) minutes 11–14 were greater than minutes 1–4 and 5–9 for Holopoint L2, and (3) HR significantly increased across all time points for Hot Squat and cycling. The simple effects tests of condition within each level of time were significant (baseline *p* = .009, minutes 1–4 *p* = .003, minutes 5–10 *p* < .001, minutes 11–14 *p* < .001). The following significant differences were revealed between games: (1) Hot Squat had greater HR than Holopoint L2 at baseline, (2) Hot Squat had greater HR than Holopoint L2 and Holopoint L3 at minutes 1–4, and (3) Hot Squat and cycling had greater HR than Holopoint L2 and Holopoint L3 at minutes 5–10 and 11–14. Holopoint L3 also had greater HR than Holopoint L2 at minutes 5–10. See Figure 2A for the HR values for each condition across time.

#### Ratings of perceived exertion

The two-way ANOVA revealed a main effect of condition (*p* < .001) and time (*p* < .001), which were superseded by a significant interaction, *p* = .003. The simple effects tests of condition within each level of time were significant for time points 6 (*p* = .040), 9 (*p* = .008), 12 (*p* = .003), and 15 (*p* < .001) minutes. Follow-up tests indicated the following significant differences: (1) Hot Squat had greater RPE than Holopoint L3 at 6 min, (2) Hot Squat had greater RPE than Holopoint L2 and Holopoint L3 at 9 min, (3) Hot Squat and cycling had greater RPE than Holopoint L2 at 12 min. (also, Hot Squat had greater RPE than Holopoint L3 at 12 min), and (4) Hot Squat and cycling had greater RPE than Holopoint L2 and Holopoint L3 at 15 min. The simple effects tests of time within each condition were all significant, *p* < .001. The following differences were found for each game: Holopoint L2: baseline < all timepoints, 3 < 9–15 min, and 6 and 9 < 15 min; Holopoint L3: baseline < all timepoints, 3 < 6–15 min, 6 < 12–15 min, and 9 < 15 min; Hot Squat: baseline < all timepoints, 3 < 6–15 min, 6 < 12–15 min, and 9 < 15 min; cycling: baseline < 3 min < 6 min < 9 min < 12 and 15 min. See Figure 2B for RPE values for each condition across time.

### Differences in actual and perceived intensity and enjoyment between games

See Table 2 for the means and standard deviations for each variable for each condition.

**Table 2:** Means and standard deviations (SD) for actual exertion variables, perceived exertion variables, and enjoyment.

	Holopoint L2	Holopoint L3	Hot Squat	Cycling
% of time in MVPA	28.9 ± 30.2	49.6 ± 33.6	78.4 ± 14.7	74.0 ± 29.7
Average %HRR	33.7 ± 7.8	39.3 ± 8.6	54.3 ± 9.6	50.3 ± 12.6
Max %HRR	47.7 ± 11.8	52.8 ± 13.2	74.1 ± 12.0	64.6 ± 15.4
Average RPE	3.7 ± 1.5	3.8 ± 1.4	4.9 ± 1.4	4.4 ± 1.3
Max RPE	5.1 ± 1.8	5.2 ± 1.8	7.2 ± 1.8	6.4 ± 2.1
Enjoyment	88.6 ± 9.8	88.9 ± 8.9	67.9 ± 14.4	63.7 ± 20.0

%, percentage; L2, level 2; L3, level 3; HRR, heart rate reserve; RPE, ratings of perceived exertion.

#### Actual intensity

##### Percent of time in MVPA (>40% HRR)

The repeated measures ANOVA was significant, *p* < .001. The significant follow-up tests indicated that Hot Squat and cycling had greater MVPA than Holopoint L2 and Holopoint L3. Holopoint L3 also had greater MVPA than Holopoint L2.

##### Average %HRR

The repeated measures ANOVA was significant, *p* < .001. The significant follow-up tests indicated the following differences in average %HRR: Holopoint L2 < Holopoint L3 < Hot Squat and cycling. Based on the %HRR values, Holopoint L2 and L3 were played at a light intensity, while Hot Squat and cycling were completed at a moderate intensity.

##### Max %HRR

The repeated measures ANOVA was significant, *p* < .001. The significant follow-up tests indicated that Hot Squat elicited higher max %HRR compared with all conditions. Also, cycling had a greater max %HRR compared with Holopoint L2. Based on the max %HRR values, the max intensity reached during Holopoint was moderate, while vigorous intensity was reached during Hot Squat and cycling.

#### Perceived intensity

##### Average RPE

The repeated measures ANOVA was significant, *p* < .001. The significant follow-up tests indicated that the participants reported greater RPE during Hot Squat compared with Holopoint at either level. No differences were evident between Holopoint L3 and Holopoint L2 in perceived exertion.

##### Max RPE

The repeated measures ANOVA was significant, *p* < .001. The significant follow-up tests indicated that the participants reported greater max RPE during Hot Squat and cycling compared with Holopoint at either level.

#### Enjoyment

The repeated measures ANOVA was significant, *p* < .001. The significant follow-up tests indicated that the participants reported greater enjoyment of physical activity during Holopoint L2 and L3 compared with Hot Squat and cycling.

### Correlations between the change in actual intensity with the change in perceived intensity within games

See Table 3 for the correlation coefficient and *p*-values. No significant correlations existed for the active VR games between the %HRR slope and RPE slope. Thus, the degree of change in actual exertion was not associated with the degree of change in perceived exertion from minute 3 to minute 12 of VR gameplay. However, the %HRR slope and RPE slope were significantly and positively correlated for cycling. Thus, greater increases in actual exertion were associated with greater increases in perceived exertion during cycling.

**Table 3:** Bivariate correlations of the degree of change in actual exertion (average %HRR) with the degree of change in perceived exertion for each VR game and exercise.

Condition	*r*-value	*p*-value
Holopoint L2	0.072	0.777
Holopoint L3	0.289	0.249
Hot Squat	0.208	0.406
Cycling	0.559	0.016*

*Note*. The degree of change is measured by the slope of the line from 3 to 12 min. L2, level 2; L3, level 3.

*Significant at *p* < .05.

## Discussion

The current study was designed to further elucidate the relationship between actual and perceived exertion during physical activity performed in active VR games. Several key findings emerged from this study. First, the actual intensity of the VR games reached moderate to vigorous, although this intensity was not maintained for the entire period of gaming. Second, increasing the difficulty level of Holopoint leads to greater actual exertion but not greater perceived exertion during gameplay. Third, the participants accurately perceived their increases in exertion during stationary cycling, but not during VR gameplay.

Based on prior research (2), we hypothesized that Hot Squat would elicit higher levels of perceived and actual exertion compared with Holopoint. We also hypothesized that increasing the difficulty level for Holopoint would increase the exercise intensity of gameplay. These hypotheses were generally supported. According to the data, approximately 75% of gameplay was spent in MVPA during Hot Squat, while approximately 50% of gameplay during Holopoint L3 was spent in MVPA, and only 30% for Holopoint L2. Prior studies have shown mixed results regarding the level of physical activity intensity obtained during Holopoint. Gomez et al. (3) demonstrated that the participants reached a moderate intensity based on METS while playing Holopoint in a customized setting, which provided a challenging difficulty level for the participants. Alternatively, Evans et al. (2) revealed that the participants played Holopoint L2 at a light intensity. The current study indicated that physical activity intensity during Holopoint is partially a function of difficulty level. It remains unknown whether further increases in Holopoint levels (higher than level 3) would result in additional gains in MVPA. Overall, our results suggest that playing these games could contribute toward the objective of obtaining 150 min of MVPA per week, with the caveat that the amount of MVPA during gameplay is likely to be less than the total duration of playtime.

In line with the parallel processing model of attention (7), a hypothesized benefit of exercising via active VR games is that the VR environment could facilitate the use of dissociative attentional strategies, in which attention is shifted from unpleasant bodily symptoms that arise during higher-intensity physical activity to the external cues of the VR game. This focus on the VR environment could then lead to an underestimation of perceived exertion during active VR games. For example, Neumann and Moffitt (8) showed that the participants running on a treadmill in a 2D VR environment (similar to watching TV) focused more attention on external task-relevant stimuli and less on internal states compared with the participants viewing neutral images while running. Additional research has shown that the participants achieve higher actual exertion during VR gameplay compared with perceived exertion for several different VR games, including Holopoint (3, 5). Our results revealed that perceived exertion was greater for Hot Squat and cycling compared with Holopoint, which was similar to the actual exertion differences. Hot Squat and cycling were rated as strong to very strong exertion, while Holopoint regardless of level was rated as moderate to strong exertion. Interestingly, even though actual exertion was higher for Holopoint L3 compared with Holopoint L2, the participants rated perceived exertion as similar between the two Holopoint levels. Thus, the more challenging levels of this game lead to greater actual exertion but not perceived exertion during gameplay. In addition, Hot Squat elicited a higher maximum actual exertion compared with cycling; however, maximum perceived exertion did not differ between Hot Squat and cycling statistically. Thus, the perceived differences in maximum exertion between Hot Squat and cycling were not as strong as the actual differences in maximum exertion. In general, these results support prior studies showing an underestimation of exertion with active VR gameplay.

In contrast to prior studies on active VR, we evaluated changes in actual and perceived exertion across time during gameplay. Supporting our hypothesis, each game increased perceived and actual exertion from baseline, with exertion generally increasing across time. Hot Squat and cycling elicited greater increases in actual and perceived exertion compared with Holopoint at both levels. The results also revealed that the participants accurately perceived their increases in exertion in our control condition, stationary cycling. Increases in actual intensity from 3 to 12 min positively correlated with increases in perceived intensity from 3 to 12 min. However, the data indicated no associations between the change in perceived and actual intensity for the VR games. While the correlation coefficients were positive for the VR games, they were small and non-significant. Thus, as the VR games progress, the participants may not accurately perceive changes in exercise intensity. It should be noted that the exercise intensity for cycling was matched to the highest intensity during VR gameplay for each individual. Thus, the participants were instructed by the experimenter to cycle faster or slower to keep HR within a target HR range. These cues, not present during VR gameplay, could have strengthened the relationship between actual and perceived exertion during stationary cycling compared with VR.

Few active gaming VR studies have evaluated the enjoyment of physical activity during VR games compared with traditional forms of exercise. The results of the present study indicated that the physical activity during Holopoint at L2 and L3 was rated more enjoyable than the physical activity during Hot Squat and stationary cycling, with no differences between the latter two conditions. Other studies have also found Holopoint to be highly enjoyable (2, 5). Moreover, McDonough and colleagues (19) evaluated physical activity enjoyment during traditional stationary cycling, a VR cycling session, and an exergame cycling session. While the physical activity intensity was not measured or standardized across the conditions, enjoyment was higher and RPE was lower for the VR cycling session compared with other cycling conditions. Finding enjoyable options for moderate-intensity physical activity participation is important because greater enjoyment or pleasure of exercise is associated with greater MVPA in the future (20, 21).

This study had several limitations. First, we suggested that the underestimation or inaccurate perceptions of exertion during VR are a result of participant immersion in the VR environment or game, which diverts attention away from unpleasant bodily symptoms. However, the current study did not actually measure attention strategies during gameplay, and this could be an important avenue for future research. In addition, based on the results of the IPAQ, the sample of the current study would be categorized as very active. The generalizability of these results to a sedentary population remains unknown. Finally, prior VR studies have used Borg's 6–20 RPE scale to measure perceived exertion, which has validated intensity categorizations based on the numerical rating given (i.e., 9–11 = light, 12–13 = moderate, etc.). The Borg CR10 RPE scale used in the present study does not have such validated intensity categorizations. Thus, we could not make intensity categorization comparisons based on RPE and %HRR data. The CR10 RPE scale was chosen based on the assumption that it would be more intuitive for the participants compared to the 6–20 scale, given that the participants had to provide ratings over a period of time without being able to see the scale.

In conclusion, our data support the notion that virtual reality may alter perceptions of exertion during physical activity and, in particular, may dampen the awareness of increases in actual exertion. Importantly, prior research indicates a negative association between perceived effort and adherence to physical activity programs (9). Thus, future research should explore whether the implementation of active VR games into physical activity programs can facilitate adherence. Furthermore, while underestimations of perceived exertion are generally assumed to have positive benefits, this phenomenon could also lead to over-exercise or over-exertion. This possibility would be important to monitor during active VR games, particularly in vulnerable populations such as older adults.

## Data Availability

The raw data supporting the conclusions of this article will be made available by the authors, without undue reservation.
